# The Invisibles: Unpaid Caregivers of the Elderly

**DOI:** 10.3389/fpubh.2014.00091

**Published:** 2014-07-21

**Authors:** Eli Carmeli

**Affiliations:** ^1^Department of Physical Therapy, University of Haifa, Haifa, Israel

**Keywords:** family caregivers, elderly population, health policy, public health, health services accessibility

## Introduction

The population of elderly individuals continues to increase and is expected to double by 2050 (1). Medical and technological improvements and developments, which extend the longevity of those with significant ailments and the push for shorter hospital stays, will result in the provision of more nursing and medical care at home. A recent report by the Alzheimer’s Association indicates that over 15 million family members are caring for a person with Alzheimer’s or another dementia (2). In 2009, more than 60 million family caregivers were providing “unpaid” care at an estimated cost of $450 billion, and the impact of care giving on the labor force will increase (3, 4). Unpaid caregivers provide the majority of long-term care for older adults. They are responsible for a variety of tasks, ranging from household chores to personal care and complex medical regimens. Care giving can result in high levels of stress, depression, and other physical and emotional manifestations known as “caregiver stress syndrome.” Caregivers need information and training in who to manage these tasks, as well as access to inexpensive community resources, and recognition that the care they provide is vital and valued. Yet, unpaid caregivers are an invisible population. The lack of strategic policy and for their support only contributes to their frustration, confusion, mismanagement, and disappointment. Therefore, a regulated system with structured procedures that can identify concerns, needs, gaps, barriers, and opportunities must be established (4).

## Who are the Unpaid Caregivers?

The role of unpaid caregiver is filled by family members, such as a spouse, child, sibling, or other relative, or a friend, or neighbor. Caregivers assist disabled, aged individuals with activities of daily living (ADL). Unpaid caregivers have heavy demands and responsibilities for caring an elderly person who is vulnerable, has poor mental health and/or physical impairments and functional decline.

## Bright Side

Becoming a caregiver for someone who is important to the caregiver has many benefits (5). In many cases, it is a privilege and a rewarding experience. Caregivers can generate a stronger bond with the person they care for. They may even find this “job” enjoyable, and a means to become a role model for the young generation. One of the 10 commandments in the Bible, to “*Respect your mother and father*” (can be interpreted as a rationale to do so, so your children will respect you). Other important advantages for caregivers are that it gives their life meaning and produces pride in their success as a caregiver. They are also able to give back to someone else. The more one gives, the more one receives. When caregivers voluntarily give of their time, energy, resources, knowledge, skills, and/or talents, they immediately set a standard of “giving” and become more sensitive and responsive to the needs of others.

## Dark Side

However, becoming a caregiver, without advance notice or preparation can have countless negative effects on a person’s life. Although care giving can be rewarding for numerous reasons, it can be demanding, overwhelming, constraining, and stressful. An individual might become a caregiver overnight without preparation, training, or skills (6), with abrupt alterations in daily lifestyle, routine, and habits. These changes can eventually trigger emotional stress and be a financial burden. Social activities might be curtailed, family and household duties might be neglected, their own health is neglected, and physical demands might cause injury. Moreover, they often feel excluded by health and social care professionals. The caregiver’s duties are immense and varied. This is known as “caregiver burden” (7). Along with other emotional burdens and psychological demands, they may be providing care for someone whom they love deeply, who has a chronic illness or a terminal disease, which can be heartbreaking. Nevertheless, they manage medications, medical appointments, and coordinate personal care. They assist with transportation, grooming, bathing, shopping, house cleaning, manage finances, etc. Schulz et al. have found empirical evidence linking patient suffering with caregiver compassion (8).

Yet, despite the tremendous responsibilities, moral obligations, and technical duties, the role of unpaid caregiver has not been formally acknowledged. There is no compensation under national social security, health insurance, welfare services, or local resources. In addition, they also need regular breaks from care responsibilities to look after their own health and well-being. This lack of recognition causes them to remain hidden or invisible (9, 10). The limited resources available for alleviating the difficulties and the demanding challenges caregivers experience are extremely problematic.

Caring for an elderly person with mental and cognitive impairment, such as dementia, Alzheimer’s disease, or physical impairment, such as Parkinson’s disease, stroke, amyotrophic lateral sclerosis, multiple sclerosis, cancer, or amputation, is exceptionally difficult. In many cases, these aged patients suffer from incontinence, risk of falling, depression, confusion, aggression, insomnia, frailty, sensory impairment (vision and hearing), poor dietary intake, and inactivity, along with social isolation, and low income. They also have minimal “participation,” as determined by the international classification of functioning, disability, and health (ICFDH), a useful model for conceptualizing disability in aging (11).

Many unpaid caregivers are adults over 55-years of age who care full- or part-time for their aged mother or father, or both. Depression, anxiety, and nervousness are common signs among many unpaid caregivers. The stress associated with the lack of support systems for aged individuals, with or without a chronic/acute illness, may result in a condition commonly referred to as “caregiver stress syndrome.” Typical symptoms include fatigue, anger, insomnia, and stomach complaints. Chronic stress can create medical problems such as high blood pressure, diabetes, and obesity; the impact of which might reduce the caregiver’s life expectancy. “Caregiver stress syndrome” is not even mentioned in the diagnostic and statistical manual of mental disorders (DSM).

## Caring for the Caregivers

First, caregivers must be recognized by formal authorities as a unique population with special concerns and needs who require special attention, consideration, and treatment. The requirements of caregivers for elderly persons are very different from the needs of those who care for children. This is mainly because elderly people are also sick, may take several prescription medications, and are more socially isolated. Thus, programs should be designed specifically for people who are caring for a loved one with cognitive decline such as Alzheimer’s disease or dementia, and emotional and psychological disturbances like depression, agitation, wandering, loneliness, unexplained apprehension, and other health and social issues.

## When the Caregiver Benefits, so Does the Care Recipient

It is well known that a satisfied worker is more productive, efficient, energetic, and is absent from work less often. The same is true with caregivers. When caregivers maintain high levels of physical and psychological performance, they provide higher motivation for those they care for. This is known as “caregiver ambiguous gain” (12). These advantages are physical and emotional/psychological and can include being more physically active, gaining self confidence, life satisfaction, and a sense of feeling needed.

## Strategy, Policy, Procedures, and Resources

Societies, especially and primarily in western and democratic countries, must encourage, support, and implement healthcare and welfare services to develop better strategies for training, treating, and dealing with the issue of unpaid caregivers (13). This requires certain actions including:

(1) Recognizing “caregiver stress syndrome” as a medical illness with a DSM/ICD-9 code.
(2) Health insurance agencies should compensate for appropriate care, provided by caregivers, which in most, if not all, cases is performed using their personal time and resources.
(3) Educating and properly training the caregivers.
(4) Online support and information on daily aspects of care giving.
(5) Telephone and email support groups for caregivers.
(6) Providing psychological counseling or psychiatric intervention for stress management. For example, forming support groups, providing home visits and one-on-one discussions.
(7) Educating employers to show more understanding of the caregivers’ working conditions, to show them empathy, and to provide some administrative support. For example, to encourage the workplace to adopt telecommuting that enable caregivers to work at home while caring for their family member; time flexibility, extended time-off as possible and if feasible.
(8) Show employers how they can benefit from supporting their employee, who provides care for a loved one. A satisfied employee is a productive employee, who is more likely to be content with his job, boss, peers, and work environment; thus, more productive, motivated, engaged, and involved.
(9) Providing caregivers with legal advice and services that will help them understand and appreciate the consequences of the client they care and help them to make rational healthcare and financial decisions, such as a living will and a living trust. Care givers are often required to make legal preparations as soon as the person they care has been diagnosed with a serious illness such as dementia.
(10) Establishing public policies and regulatory mechanisms to protect the rights and guide the role of caregivers, by developing and maintaining operational principles and procedures that address the holistic management of unpaid caregivers. Moreover, such a mechanism shall develop and maintain fiscal control and funding that will be dedicated to the benefit of the caregivers not by paycheck or salary, but for education and guidance.
(11) Additional empirically validated research and methodology are needed to investigate the needs, concerns, and expectations of caregivers and the effectiveness of their interventions, as also suggested by Zarit and Femia (14).

## Summary

At a time when hospitals discharge patients earlier, elderly individuals are living longer, and with many chronic illnesses, more family members and friends are caring for loved ones at home. As people continue to age and receive multifaceted health care services at home, concern has arisen about the availability of unpaid family caregivers and their ability to combine employment with care giving. Unpaid caregivers often operate in a reality of inadequate support systems and lack of information and skills. Yet, they manage multiple roles, duties, and responsibilities for supporting the needs of older adults. Often, unpaid caregivers find themselves having to perform unfamiliar and unusual tasks. These may include giving medications, assisting with bathing, dressing, meals and intimate personal hygiene, dealing with transportation, billing and medical appointments, and performing housekeeping and nursing activities (15).

Despite the fact that unpaid caregivers have diverse cultural norms, knowledge, skills, willingness, and attitudes, they all have common concerns and needs. They need guidance, someone to listen to them, and a place they can share concerns, express needs, and receive answers. Conceptual and methodological approaches that recognize, acknowledge, and support the complexities of unpaid care giving are needed. Greater access to formal care for unpaid caregivers, a national strategy, local and workplace policies, and other supportive systems must be established.

## Conflict of Interest Statement

The author declares that the research was conducted in the absence of any commercial or financial relationships that could be construed as a potential conflict of interest.
